# Value-based pricing of cognitive behavioral therapy for depression in primary care: an economic evaluation

**DOI:** 10.1186/s12913-024-10653-5

**Published:** 2024-03-08

**Authors:** Afschin Gandjour

**Affiliations:** Frankfurt School of Finance & Management, Adickesallee 32-34, Frankfurt am Main, 60322 Germany

**Keywords:** Value-based pricing, Psychological treatment, Decision-tree model

## Abstract

**Objectives:**

Value-based pricing (VBP) determines product prices based on their perceived benefits. In healthcare, VBP prices medical technologies considering health outcomes and other relevant factors. This study applies VBP using economic evaluation to provider-patient communication, taking cognitive behavioral therapy (CBT) for adult primary care patients with depressive disorders as a case study.

**Methods:**

A 12-week decision-tree model was developed from the German social health insurance system’s perspective, comparing CBT against the standard of care. The influence of an extended time horizon on VBP was assessed using a theoretical model and long-term data spanning 46 months.

**Results:**

Using a willingness-to-pay threshold of €88,000 per quality-adjusted life year gained, the base-case 50-minute compensation rate for CBT was €45. Assuming long-term effects of CBT significantly affected the value-based compensation, increasing it to €226.

**Conclusions:**

This study showcases the potential of applying VBP to CBT. However, significant price variability is highlighted, contingent upon assumptions regarding CBT’s long-term impacts.

## Introduction

Value-based pricing (VBP) sets product prices based on perceived consumer benefits and finds applications across various sectors including healthcare, manufacturing, logistics, and telecommunications. In healthcare, it determines medical technology prices according to health benefits and other factors. Economic evaluation-based VBP requires an explicit willingness-to-pay threshold, predominantly discussed in pharmaceutical pricing. Such a VBP approach awards the full innovation value to the manufacturer [1]. Notably, VBP using economic evaluation can also dictate provider-patient communication prices, essentially pricing providers’ communicative time with patients. This approach aligns provider reimbursement with mental and behavioral health outcomes [2]. This means setting health professionals’ salaries according to anticipated patient health benefits and associated cost savings. Unlike risk-sharing, a payment model based on actual outcomes [3], VBP can rely on expected outcomes.

For effective VBP in provider-patient communication, health benefits derived from communication must be distinguishable from benefits via other means, like drug prescriptions. Cognitive behavioral therapy (CBT) meets this criterion by delivering health benefits solely through communication. This study aims to apply VBP to provider-patient communication using CBT for adult primary care depression patients, comparing it to standard care. The objective is to ascertain health professional salaries based on the health benefits and cost savings facilitated by CBT. Germany serves as the application’s focal country, where the 2019 significant psychotherapy price increase responded to the previously slower psychotherapist income growth [4]. Currently, the reimbursement for a 50-minute one-to-one long-term therapy session stands at €103.87, inclusive of overhead costs (as per fee no. 35425 of the ambulatory physician fee schedule of the social health insurance (SHI) [5]).

As an indication for CBT, depressive disorders such as major depression, dysthymic disorder, and unipolar depression were considered. Major depression is prevalent in Germany, with a point prevalence in 2014/15 estimated at 10% [6]. Additionally, the economic impact of major depression is significant, with costs in Germany estimated at €8.7 billion in 2015, excluding productivity losses [7].

## Methods

As outlined in the introduction, this study aimed to determine the salary of health professionals based on the health benefits and cost savings achieved through CBT in treating depressive disorders. Within a VBP framework that employs economic evaluation, the maximum price or reimbursement for CBT is determined by setting the incremental cost-effectiveness ratio (ICER) against a less effective treatment equal to the cost-effectiveness threshold, represented as *λ*:

1 $$ \frac{c}{h}=\frac{p+b}{h}= \lambda.$$

Here, $$ c$$ represents incremental costs, $$ h$$ stands for incremental health benefits, $$ p$$ is the maximum acceptable incremental price of CBT, and $$ b$$ presents incremental costs induced by CBT (e.g., costs savings from preventing depression-related morbidity and life extension costs from preventing premature deaths due to suicide).

By rearranging Eq. (1), the maximum acceptable price of CBT is derived (see [8]):

2 $$ p=\lambda{h-b}.$$

As illustrated in Eq. (2), the price or compensation rate of CBT has a linear relationship with willingness to pay. To compare with the current rate, the compensation rate of CBT is associated with a time unit, resulting in:

3 $$ \dot{p}=\frac{\lambda{h-b}}{t}.$$

Here, $$ \dot{p}$$ represents the compensation rate for CBT per unit of time, while $$ t$$ indicates the duration of care provision.

Attributing health benefits and downstream costs of CBT to health professionals becomes intricate when considering subsequent treatment decisions. This is especially pertinent when evaluating the depression’s lifetime course since treatment types and intensity might vary. For instance, the depression guideline by the German Association for Psychiatry, Psychotherapy, and Psychosomatics [9] suggests patients who have undergone acute depression psychotherapy to continue with a less intensive maintenance psychotherapy over 8 to 12 months. Each new decision, like the introduction of maintenance psychotherapy, necessitates a re-evaluation of the value-based compensation. A pertinent query arises: should the health benefits and subsequent costs of maintenance psychotherapy and subsequent therapy decisions be attributed to the initial CBT? Such an attribution aligns with economic evaluation standards, which generally mandate health benefits and costs extrapolation beyond a trial’s time horizon [10]. However, when subsequent treatments are also priced based on VBP, the costs and health benefits of those treatments and the initial psychotherapy effectively offset each other. Formally, when ICERs of following (psycho-) therapies also align with the threshold, differences in costs and health benefits between initial psychotherapy and subsequent therapies are mainly due to a proportionality factor, denoted as *x*:

4 $$ \frac{c}{h}=\frac{xc}{xh}=\lambda.$$

When comparing subsequent (psycho-)therapy to its comparator, its effectiveness may or may not surpass that of the initial psychotherapy relative to its comparator. Therefore, $$ x$$ is defined within the range [0, ∞]. When the effectiveness levels are equivalent, $$ x$$ is set to 1. It is worth noting that $$ x$$ facilitates the inclusion of a sequence of (psycho-)therapies, contingent on the time horizon’s length. Formally:

5 $$ x=\sum _{i=1}^{n}{x}_{i}$$

Here, $$ n$$ signifies the count of subsequent (psycho-)therapies. Another crucial aspect is that $$ x$$ can encompass a time discount factor for both costs and health benefits. Consequently, the model is equipped to consider delays in subsequent (psycho-)therapies. Moreover, $$ x$$ has the capacity to integrate probabilities of later (psycho-)therapies within an indeterminate framework.

Factoring in the costs and health benefits of one or more follow-up therapies into the ICER of the preliminary CBT gives, based on Eq. (4):

6 $$ \frac{c+xc}{h+xh}=\lambda.$$

Basic arithmetic reveals that the costs and health benefits from subsequent (psycho-)therapies neutralize:

7 $$ \frac{c\left(1+x\right)}{h(1+x)}=\frac{p+b}{h}=\lambda.$$

As per Eq. (7), integrating costs and health benefits from later (psycho-)therapies (and other treatments) does not alter the ICER of the initial psychotherapy. In simpler terms, if subsequent therapies are priced in alignment with the threshold ICER, the cost of the original psychotherapy remains unaffected.

An opposing viewpoint might suggest that follow-up treatments could encompass generic antidepressants or other treatments with an ICER below the threshold. From the payer’s perspective, compensating health professionals with a higher salary solely because subsequent treatments are generic or affordably priced might be seen as contentious. This could inadvertently allocate savings from more affordable medications to the health professional. A parallel argument was presented regarding the value-based pricing of curative therapies, contending that “innovators should not reap all of the financial rewards related to cost offsets generated by a cure” ([11], p. 659). Pearson et al. [11] asserted that “the price of a cure for one person should not be worth more than that for another just because one person’s condition is currently very expensive to treat” (p. 658).

**Fig. 1:**
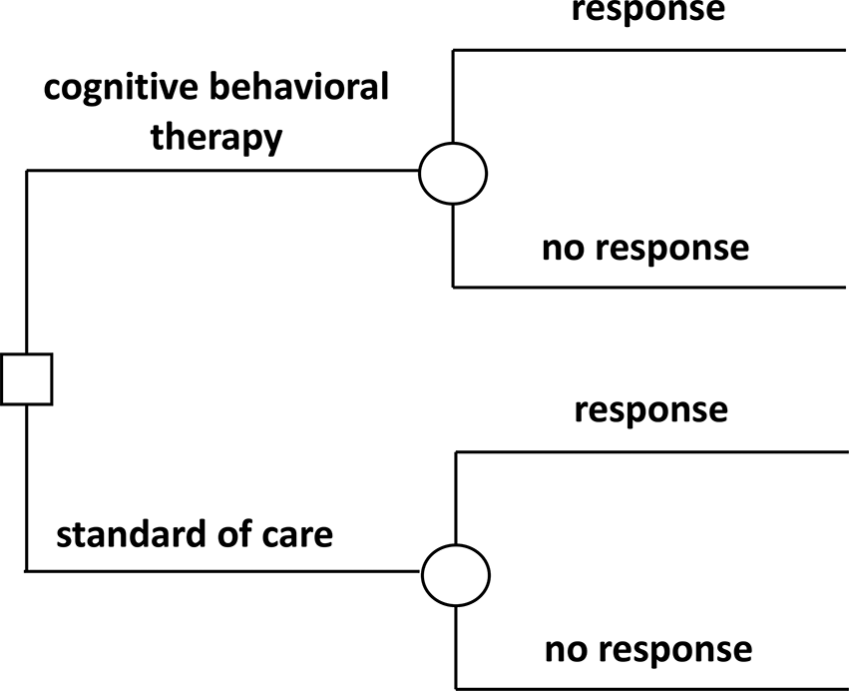
Decision tree illustrating treatment options and associated probabilistic events within the model

Given the aforementioned deliberation, the decision model can be streamlined to a single treatment choice following a singular clinical occurrence, namely the depressive disorder. This can be visualized through a decision tree (refer to Fig. 1). The base-case time horizon was aligned with the treatment duration at 12 weeks (refer to Data). With a time horizon less than a year, both cost and outcome discount rates were designated as 0%. A sensitivity analysis also took into account evidence pertaining to the prolonged efficacy of CBT, spanning 46 months.

As a measure of health benefits, the study employed quality-adjusted life years (QALYs). QALYs serve as a ‘common currency’ allowing for comparisons across interventions for all diseases. They combine the number of life years with the strength of preference for various health states. The strength of preference is quantified on a scale where 0 represents death and 1.0 signifies perfect health. Accordingly, in this research, VBP was rooted in a cost-utility analysis.

QALYs were determined based on the number of avoided remissions and suicides. To estimate avoided suicides, a preliminary back-of-the-envelope analysis was conducted. Owing to its inherent uncertainties, this analysis is provided as part of the sensitivity analysis. Specifically, the incidence of suicide in major depression over the specified time horizon was calculated and then multiplied by the effect of CBT on suicide attempts and the remaining quality-adjusted life expectancy for an averted suicide case.

The model adopted the perspective of the German SHI. Therefore, it encompassed direct medical costs but excluded direct non-medical costs, such as transportation. This is because such costs are typically not covered by the SHI in relation to CBT.

### Data

Effectiveness of CBT for depression in adult primary care patients was ascertained from a PubMed literature search conducted in March 2023. The following search strategy was employed: *“Cognitive Behavioral Therapy“[MAJR] AND (“Depression/therapy“[MAJR] OR “Depressive Disorder/therapy“[MAJR]) AND “Primary Health Care“[MAJR] Filters: Meta-Analysis*. The meta-analysis chosen for this study was the one with the most recent literature search, conducted by Santoft et al. [12]. This meta-analysis incorporated 34 randomized controlled trials (RCTs). For inclusion, patients either had to (i) meet diagnostic criteria for a unipolar depressive disorder, such as DSM-IV major depressive disorder, (ii) score above a recognized cut-off for depression, or (iii) present depressive symptoms. There were no restrictions regarding the number of prior depressive episodes in this meta-analysis. Online treatments were considered if they also involved support from a clinician. However, specifics regarding the qualifications of the clinician (e.g., psychologist, psychiatrist, etc.) were not provided. The most frequently employed comparator was “treatment as usual,” which was used in 29 out of the 46 control groups. This encompassed other psychological treatments, antidepressant drugs, and no treatment. Other comparators involved antidepressant drugs, alternative psychological treatments, waiting lists, and placebos (either psychological or pharmacological) as detailed in Santoft et al. [12]. The study emphasized 17 specific RCTs that provided a summary estimate on remission. Here, remission was defined as the proportion of patients who no longer met the criteria for depression or who scored below a recognized threshold for depression. Using remission provided a binary representation (yes/no) of the clinical outcome of CBT. This binary approach facilitated the assignment of a categorical utility score, as available from the existing literature.

Using the ‘assessment of multiple systematic reviews’ (AMSTAR) measurement tool [13], only 5 of the 11 criteria were determined to be adequately addressed. Notably, the heterogeneity of the underlying trials, which can be partly attributed to the diverse comparators, raises concerns. Furthermore, Santoft et al. [12] identified a high risk of bias in the underlying RCTs, even when overlooking unblinded participants and personnel — a challenge intrinsic to the nature of the treatment.

The meta-analysis also delved into various moderators, revealing that baseline severity of depression had no significant correlation with effect size using a continuous measure (Hedges’ g). Nevertheless, the influence of different baseline utility scores was explored as part of the sensitivity analysis.

A treatment duration of 12 weeks was presumed, mirroring the median time to primary endpoint among the 34 RCTs featured in the meta-analysis. However, this detail was unavailable for the subset of 17 RCTs providing a summary estimate on remission [12]. This treatment duration aligns well with recommendations by the German Association for Psychiatry, Psychotherapy, and Psychosomatics [9] for acute therapy of depression, set at 6 to 12 weeks. Session numbers and durations in the model (15 sessions lasting 50 min each) were sourced from an official directive by the German Federal Joint Committee [14]. This directive stipulates 2 to 4 preliminary individual sessions of 50 min each, followed by up to 24 individual sessions lasting at least 25 min for acute treatment. In the German SHI system, the compensation for two 25-minute sessions equates to one 50-minute session. Similarly, the National Institute for Health and Clinical Excellence ([15], p. 298) advocates that “for all people with depression having individual CBT, the duration of treatment should typically be in the range of 16 to 20 sessions over 3 to 4 months.” This lower boundary (16 sessions over 3 months) harmonizes with the model’s input. Although maintenance therapy post-acute treatment (i.e., after 6 to 12 weeks) is deemed useful [9], the model does not project costs and health advantages beyond the median RCT duration. As demonstrated in the prior section, when maintenance therapy is also subject to VBP, its costs and benefits offset each other.

For the base-case, an absolute reduction of remissions by 9.9% was applied, derived from Santoft et al.’s [12] summary estimate for the number needed to treat (NNT) of 10.08 (with a remission rate in the control conditions at 35%). Refer to Table 1 for a complete list of input parameters. The preference weights originated from EQ-5D scores (Dutch tariff), based on an analysis of patient-level data from 10 RCTs with 1629 participants [16]. Mild depression was excluded, as it was also omitted from the cost analysis source ([17], p. 379), preventing a potential disparity between utility and cost data. To integrate preference weights of both moderate and severe depression in the base-case, proportions were sourced from a survey of primary care patients in Germany [18]. This survey indicated that twice as many patients were diagnosed with moderate depression as compared to severe depression [18]. The diagnostic process relied on the patient self-reported Depression Screening Questionnaire. The survey’s prevalence data align with the findings from health insurance data in a report by the Institute for Quality and Efficiency in Health Care (IQWiG) ([17], p. 547). In a sensitivity analysis, the model accounted for different depression levels at baseline by applying the relevant preference weights. Comparators other than “treatment as usual” were not taken into account due to the unavailability of corresponding remission rate data in Santoft et al. [12].

**Table 1:** Base-case values and ranges (95% confidence intervals)

Variable	Mean (range)	Reference
Absolute reduction of remission rate (%)	9.9 (2.7–17.2)	[12]
Preference score		[16]
- Remission of depression	0.73 (0.69–0.77)	
- Moderate depression	0.51 (0.47–0.55)	
- Severe depression	0.37 (0.33–0.41)	
Cost per CBT session (€)	103.87	[5]
Cost of 100 mg fluvoxamine over 12 months considering mandatory rebates for the SHI (€)	70.96	[19]
Direct costs of depression over two months excluding costs of psychotherapy (€)	173.30	[17]
Indirect costs (€)	0 (200.64)	[17]
Number of CBT sessions	15	[14]
Proportion of patients with moderate depression (%)	68 (61–75)	[18]
Proportion of depressive patients receiving psychotherapy (%)	10 (6–15)	[18]
Proportion of depressive patients receiving antidepressants (%)	26 (19–33)	[18]

CBT = Cognitive behavioral therapy, SHI = social health insurance

The report by IQWiG [17] was used to source inpatient, outpatient, and medication costs associated with depression. This document consolidated cost data from multiple sources, including the most prominent German health insurer during the time of the report’s release (Barmer GEK). The presented cost data was solely associated with depression treatment, excluding unrelated costs. The model accounted for the cost of non-responders by considering the cost of no response under placebo treatment, as detailed in IQWiG's report, while excluding psychotherapy and medication expenses to prevent double counting. In a sensitivity analysis, indirect costs stemming from work productivity loss were included based on IQWiG’s [17] report. Incorporating these indirect costs offers an insight into the most significant impact of productivity loss from a payer’s standpoint since sickness funds only cover extended sick leaves after six weeks.

In the Santoft et al. [12] meta-analysis, the most common comparator was treatment as usual, which encompassed various types of comparators. For this reason, the costs of current care were assigned to the comparator arm using empirical data on the utilization of different treatments for moderate and severe depression in primary care patients in Germany [18]. The fee for CBT (no. 35425) from the ambulatory SHI physician fee catalogue, which pertains to general behavioral therapy, was applied for psychotherapy costs [5]. For the costs of antidepressants, the least expensive drug (fluvoxamine) deemed suitable for treating moderate and/or severe depression was used [19], introducing potential bias against CBT. Costs of diagnostic workup before treatment (fee no. 35151) [5] were not included, as they would be applicable to both comparators and would therefore offset.

A sensitivity analysis was conducted to estimate the health benefits of CBT in suicide prevention. This utilized results from a meta-analysis of RCTs comparing CBT to standard care regarding repeated suicide attempts, primarily in depressed patients [20]. The same meta-analysis was employed in a cost-effectiveness modeling study for adult primary care patients in the U.S [21]. 

In Germany, the prevalence of major depression based on the cutoff score of the eight-item Patient Health Questionnaire depression scale (PHQ-8) was 10.1% in the adult population (≥ 18 years) in 2014/15 [6]. Furthermore, the number of suicides in Germany was 10,076 in 2013 [22]. In agreement with prior literature, we attributed 50% of suicides to major depressive disorders [23]. We multiplied the reduction in suicides with the remaining quality-adjusted life expectancy at age of 45 years, utilizing data from a published patient-level decision model that simulates transitions between major depressive episodes and remission [24].

**Fig. 2:**
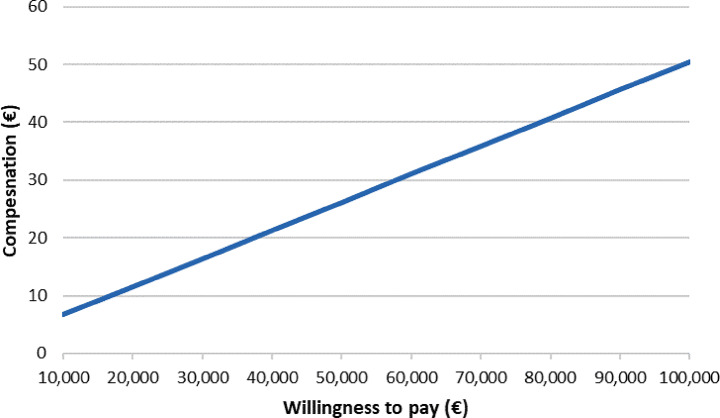
Sensitivity analysis of the reimbursement rate for a 50-minute cognitive behavioral therapy session in relation to the willingness to pay per quality-adjusted life year

In the sensitivity analysis, evidence on the long-term effectiveness of CBT was considered to account for the chronic relapsing nature of depression [25]. In an RCT with a median follow-up of 46 months [25], the remission rate of 12 to 18 CBT sessions, in addition to usual care, was 28% compared to 18% in the control group at 46 months. This absolute reduction is comparable to the results reported by Santoft et al. [12] at 12 weeks. The treatment effect appeared consistent, as evident from Fig. 2 of the publication [25]. Based on the associated analysis of the trial, which determined that “health-care costs were very similar for the duration” ([25], p. 142), no additional savings were modeled. Should a cost-effectiveness analysis model other (psycho-)therapies, both costs and health benefits of subsequent (psycho-)therapies would neutralize each other, as depicted in Eq. 7.

All costs underwent inflation adjustment to 2019 euros using the general German Consumer Price Index. The cost-effectiveness threshold was established at €88,000 per QALY gained, mirroring health opportunity costs within the German SHI [26].

### Sensitivity analysis

In deterministic one-way analyses, parameter uncertainty was assessed by varying individual input parameters susceptible to variation, one at a time, using the boundaries of the 95% confidence interval (Table 1). Typically, to evaluate the effect of simultaneous changes in multiple variables on the target outcome variable, a Monte Carlo simulation, a form of multivariate sensitivity analysis, is conducted. However, due to the predominant influence of a single variable, specifically the duration of the treatment effect (refer to Results), executing a Monte Carlo simulation was not deemed valuable for offering supplementary insights.

## Results

In the base-case analysis, the incremental QALY gain stands at 0.007, taking into account a 12-week time horizon, an NNT of 10, and a utility gain of 0.26 under remission. With incremental costs amounting to €1308, predominantly influenced by the official cost of a CBT session (€103.87) and its duration (15 sessions), the ICER calculates to €207,995 per QALY gained. Given a willingness-to-pay (WTP) threshold of €88,000 per QALY gained, the value-based fee for a 50-minute CBT therapy session should not exceed €45. The variation of this rate in relation to WTP is depicted in Fig. 2.

**Fig. 3:**
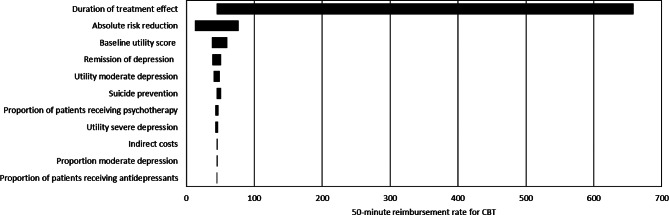
One-way sensitivity analysis of the reimbursement rate for a 50-minute cognitive behavioral therapy (CBT) session, based on a willingness to pay of €88,000 per quality-adjusted life year. Variables are arranged in order of their impact on the reimbursement rate

Within the one-way sensitivity analysis, the duration of CBT remission emerged as the input parameter exerting the most significant impact on the reimbursement rate, as illustrated in Fig. 3. The rate escalates to a maximum of €226, presuming a continuous treatment effect spanning 46 months. The related ICER computes to €13,565 per QALY gained.

In reference to the NNT for depression remission as proposed by Santoft et al. [12], the derived NNT for suicide prevention is 16,409. Thus, even when applying the remaining quality-adjusted life expectancy for an averted suicide case, the surge in QALYs is only 0.001. Incorporating this QALY gain pushes the average value-based fee for a 50-minute session from €45 to €51.

## Discussion

This study employs a decision tree model to determine the salary of health professionals based on the value they deliver. Taking CBT as an exemplar, this paper underscores the feasibility of allocating value-based remuneration but also points out potential challenges. Assessing the rate of remission with and without CBT, the study’s base-case outcome indicates that the value-based compensation for a 50-minute CBT session, which is €45 based on a WTP threshold of €88,000 per QALY, considerably undercuts the prevailing rate (€104). However, when factoring in a potential extended impact on remission (spanning 46 months), this observation is inverted, suggesting a value-based reimbursement rate that is double the current rate.

The ICER computed for CBT stands at €207,995 per QALY gained in the base-case. Such a figure warrants cautious interpretation due to the limited 12-week time horizon. As highlighted, taking into account a prolonged duration of effect (stretching over 46 months) markedly bolsters cost-effectiveness, attributed to a more significant QALY gain, even in the absence of supplementary savings (the impact on savings in the accompanying analysis of the pertinent long-term trial was minimal [25]). Still, data on prolonged efficacy stems from a solitary RCT and thus invites circumspection.

Additionally, the research prompts consideration of the extent to which policymakers should differentiate reimbursement levels—possibly by CBT type or initial depression severity—or opt for a standardized lump-sum reimbursement. There is an inherent balancing act between refining reimbursement levels to echo the genuine worth of CBT and navigating the increased administrative intricacies and the amplified ambiguity in valuing services. Importantly, sidestepping undesirable treatment incentives, such as cream skimming, remains a challenge, regardless of whether one adopts a nuanced or a lump-sum payment approach.

Past modeling evaluations concerning the cost-effectiveness of depression treatment in Germany [17, 27–32] have employed existing prices to compute cost-effectiveness ratios rather than establishing prices grounded on cost-effectiveness analysis (in the context of VBP). To the extent of current knowledge, a mere three of these studies have explored the cost-effectiveness of psychological treatment [30–32]. Although the analysis by Biddle et al. [31] also encompasses CBT for psychological treatment, it poses a distinct research question and differs substantially in multiple aspects, including the duration of the time horizon, magnitude of comparator costs, the NNT of CBT, and the count of CBT sessions. Another recently released study [32] that takes into account CBT, deviates in terms of its research inquiry and time horizon. Utilizing a protracted time horizon, as deployed in these studies, is fitting for analyzing the acceptability of an intervention’s current reimbursement rate, but does not furnish supplementary data when determining the value-based reimbursement rate, as outlined in Methods.

Besides the treatment duration, a pivotal determinant in computing the ICER is the NNT of CBT, as revealed in the sensitivity analysis. The NNT employed in this model originates from a recently conducted meta-analysis [12]. To verify the credibility of the NNT, especially given the identified limitations of this meta-analysis discussed in the Methods section, a comparison is made with NNTs computed in alternate studies. Notably, diverging from the majority of meta-analyses on psychotherapy, which determine the NNT based on a transition in depressive symptoms gauged on a continuous depression scale (utilizing metrics such as Hedges’ g), the meta-analysis by Santoft et al. [12] leverages cutpoints that bifurcate continuous depression scale scores [12]. Potential variations might arise regarding CBT delivery settings (primary vs. specialist care) and initial depression severity. Another meta-analysis executed recently [33], which spanned literature until January 2018, identified an NNT of CBT at 6.14 for a transition in depressive symptoms, corresponding to a Hedges’ g effect size of 0.35 (irrespective of the care context and grounded on 55 comparative RCTs with a low risk of bias). This outcome situates itself between the Hedges’ g effect sizes for primary and specialist CBT settings highlighted in the Santoft et al. [12] meta-analysis (0.22 and 0.43, respectively) and thereby indirectly affirms the elevated NNT employed in this study for primary care.

The parsimonious nature of the model means it rests on a relatively few foundational assumptions. While certain assumptions potentially pushing towards higher reimbursement for CBT have been touched upon earlier, there are also factors within the model that may lead to an inadvertent underestimation of the cost-effectiveness of psychological treatment for depressive disorders, and consequently, the remuneration for health professionals.

One such factor is the model’s lack of explicit consideration for treatment discontinuation. Some patients might stop their depression treatment prematurely, leading to no incurred intervention costs. This omission is attributed to the unavailability of compliance data from the Santoft et al. [12] meta-analysis. However, it is worth noting that compliance rates are indirectly accounted for within the effectiveness data, which might, in turn, reduce the perceived treatment efficacy.

Furthermore, the study solely zeroes in on costs that are directly associated with depression, adhering to the framework provided by the data source [17]. However, depression is also linked with elevated healthcare costs in areas not directly tied to the ailment itself, as suggested by Simon et al. [34]. If these indirect costs were integrated, the cost-effectiveness profile of depression treatment could likely appear more favorable. While the study’s cost data spans a 2-month duration, which may lead to a potential underestimation of cost-effectiveness, the derived 2-month cost estimate (€173) aligns closely with a 3-month estimate from another German study [35]. The latter integrates psychotherapy and medication costs, leading to a total of €195 over three months.

Another assumption worth highlighting is the model’s premise that treatment effects are universally replicable, irrespective of settings and providers. However, in real-world scenarios, certain health providers might exhibit a superior knack for delivering CBT. Moreover, some may promote lifestyle changes in conjunction with CBT, potentially amplifying the positive outcomes.

The study’s reference to the long-term findings by Wiles et al. [25] is based on data from patients diagnosed with treatment-resistant depression. Extrapolating these findings to other types of depression might not be wholly accurate. Lastly, an aspect to be cautious about is the determination of QALYs. If based on health state evaluations by depressive patients, the calculation might be skewed due to the influence of a maximal endurable time, as suggested by Weyler et al. [36].

The presented VBP approach in this manuscript is universally applicable and can be extended to any geographical context. In healthcare frameworks characterized by multiple payers, where individual payers determine their own WTP threshold, the resultant value-based compensation might exhibit variations across different payers.

In contrast to VBP, several alternative methods exist for determining provider payment communication in healthcare settings. These alternatives include fee-for-service models, capitation, pay-for-performance schemes, and bundled payments. Each of these approaches carries its own set of advantages and challenges. Fee-for-service models, for instance, compensate healthcare providers based on the volume of services delivered, which can lead to overutilization and rising healthcare costs. Capitation, on the other hand, provides fixed payments per patient, encouraging cost containment but potentially compromising the quality of care. Pay-for-performance schemes reward providers based on specific clinical outcomes, promoting quality but sometimes neglecting broader patient-centered care. Bundled payments group together services related to a specific condition or procedure, offering potential cost savings but posing challenges in determining fair pricing for bundled services.

In contrast, VBP as demonstrated in this study strives to align provider reimbursement with the value delivered to patients, considering both the cost-effectiveness of interventions and the impact on patient outcomes. While VBP may present administrative complexities and require careful consideration of thresholds and data sources, it offers a promising avenue to balance quality, cost-effectiveness, and patient-centered care in healthcare remuneration.

Subsequent studies might explore the VBP concept across various indications for CBT or even extend to other therapeutic modalities that hinge on the communication dynamics between the provider and the patient. Tailoring the approach to different therapeutic indications might necessitate the adoption of varied modelling techniques, like Markov models. Another promising area for exploration might be the exploration of potential barriers or resistance encountered when adjusting the reimbursement levels for existing market services, exemplified by CBT for depression, post the VBP application. Drawing from this study, the influence of trial duration on both the ICER and the value-based pricing underlines another avenue for research: how to appropriately weigh evidence derived from the most prolonged trial in contrast to evidence procured from other trials. However, before charting definitive policy directions or widely implementing VBP premised on economic evaluations to determine equitable remunerations, these facets warrant thorough examination.

## Data Availability

All data are contained within the manuscript.

## Declarations

All methods were carried out in accordance with relevant guidelines and regulations.
